# BEYOND PHYSICAL ACTIVITY: ALASKA NATIVE CONCEPTS OF PHYSICAL HEALTH, MOVEMENT, AND SUCCESSFUL AGING

**DOI:** 10.1093/geroni/igae098.1128

**Published:** 2024-12-31

**Authors:** Maja Pedersen, Steffi Kim, Lena Thompson, Jordan Lewis

**Affiliations:** University of Montana Missoula, Missoula, Montana, United States; University of Alaska, Anchorage, Alaska, United States; University of Minnesota Duluth, Duluth, Minnesota, United States; University of Alaska Fairbanks, College of Indigenous Studies, Fairbanks, Alaska, United States

## Abstract

Physical health is a key component of Western-centered models of successful aging. Physical movement in the form of exercise and functional activities are described in the literature as strategies to maintain health through reduced risk for falls, pain, and chronic disease, and to increase quality of life. However, physical health may not be consistently conceptualized across cultures, and few studies have explored this topic among Alaska Native (AN) older adults. We used thematic analysis to evaluate physical health in qualitative interviews with 162 AN Elders across the five regions of Alaska, collected over 16 years. Five overall themes were identified. (1) Elders described recognizing physical changes in their bodies, including gray hairs, or wrinkles. These changes were often seen as signs associated with wisdom. (2) Elders described that, despite physically slowing down, they adjusted tasks to do as much as possible, and found small ways to include daily movement. (3) Elders emphasized the importance of caring for oneself when young, including avoiding substance use. (4) Elders recommended addressing health challenges with a doctor, rather than ignoring them. (5) Elders described physical health as closely tied to living one’s Native ways, including physical activity through a subsistence lifestyle (e.g., gathering, hunting, fishing), relationship with the land, and benefits from eating Native foods. Overall, Elders found ways to preserve their physical health during challenging times and adjust to new norms. These findings can guide the development and tailoring of interventions designed to promote physical health among AN Elders for relevance and impact.

